# Scaling Up HIV Self‐Testing in Africa: Insights From National Programmatic Data in Eight Countries

**DOI:** 10.1002/jia2.70120

**Published:** 2026-04-29

**Authors:** Adrien Allorant, Anne Bekelynck, Aliza Monroe‐Wise, Carlota Baptista da Silva, Thato Chidarikire, Olanrewaju Edun, Leonid Joaquim, Christine Kisia, Joseph Larmarange, Johnson John Lyimo, Juma McOllogi James, Christine Musanhu, Getrude Ncube, Isabel Sathane, Arlette Simo‐Fotso, Geoffrey Taasi, Cheryl Case Johnson

**Affiliations:** ^1^ Department of Social Statistics and Demography University of Southampton Southampton UK; ^2^ Global HIV, Hepatitis and STI Programmes World Health Organization Geneva Switzerland; ^3^ WHO Pretoria South Africa; ^4^ School of Public Health, Imperial College London London UK; ^5^ WHO Maputo Mozambique; ^6^ WHO Nairobi Kenya; ^7^ French Institute for Demographic Studies, INED Aubervilliers France; ^8^ WHO Dar es Salaam Tanzania; ^9^ Ministry of Health, The National AIDS, Sexually Transmitted Infections and Hepatitis Control Program (NASHCoP) Dodoma Tanzania; ^10^ Ministry of Health Harare Zimbabwe; ^11^ Global Programs for Research and Training Maputo Mozambique; ^12^ Centre Population & Developpement, Universite Paris‐Cite, IRD Inserm Paris France; ^13^ Ministry of Health Kampala Uganda

**Keywords:** diagnosis, HIV/AIDS, HIV testing, linkage to treatment, routine data, self‐test

## Abstract

**Introduction:**

Evidence from routine, national programme data on HIV self‐testing (HIVST) scale‐up is limited. This study examines HIVST scale‐up in eight African countries, describing how HIVST has been integrated into testing strategies and how testing coverage, test positivity, and linkage to antiretroviral therapy (ART) have evolved.

**Methods:**

We conducted a retrospective descriptive analysis of national programme data from January 2019 to December 2023 across Kenya, Lesotho, Malawi, Mozambique, South Africa, Tanzania, Uganda and Zimbabwe. Data were disaggregated by quarter and subnational district. Indicators included HIVST kits distributed, conventional testing volumes, new HIV diagnoses and new ART initiations. We derived testing rates, testing positivity, ART linkage, and stability of HIVST distribution by district and over time.

**Results:**

HIVST scale‐up varied across countries. By the most recent quarter, HIVST accounted for 63% of total testing in Lesotho, 19%–25% in Malawi and Zimbabwe, but <15% in Kenya, Tanzania, Uganda and South Africa. In Malawi, Lesotho and Zimbabwe, large volumes of HIVST partially offset declines in conventional testing during the COVID‐19 pandemic. HIVST remained modest (<15% of total tests) in Kenya and Tanzania. In Mozambique, both conventional testing and HIVST expanded. In South Africa, conventional testing remained high after COVID‐19, while HIVST expanded slowly. Despite divergent trajectories, new HIV diagnoses and ART initiations remained stable in most settings, indicating programmes adapted to maintain case‐finding even as testing volumes shifted.

**Conclusions:**

This descriptive analysis shows HIVST has been scaled to different degrees, with its contribution to overall testing shaped by national contexts, and distribution models. Interpretation is constrained by incomplete reporting, the inability to identify kits used out of kits distributed and distinguishing first‐time from repeat testers. These findings can guide optimizing HIV testing services, an essential step towards meeting global HIV targets and ending AIDS by 2030.

## 1 Introduction

Despite considerable progress over the past decade, approximately 14% of people living with HIV in eastern and southern Africa did not know their status in 2023 [1], with men, adolescents, young adults and key populations disproportionately underserved [2]. Closing these gaps is imperative for reaching global HIV targets.

HIV self‐testing (HIVST) has emerged as a user‐centred, low‐barrier option to increase access to testing by bypassing many obstacles associated with traditional testing models, such as stigma, discrimination and logistical constraints [3]. This strategy empowers individuals to take greater control of their health while also offering opportunities to reach those less likely to engage in conventional testing, particularly through distribution in high‐risk social and sexual networks [4, 5]. Early studies demonstrated that HIVST is accurate, acceptable, safe and effective at increasing testing uptake [6, 7, 8]. Following the World Health Organization (WHO) recommendations in 2016 [9], expanded in 2024 [10], 108 countries have adopted national guidelines, with 71 routinely implementing HIVST, predominantly in Africa [11]. Implementation approaches, however, vary by country; Mozambique offers HIVST primarily through community distribution, Zimbabwe employs both community‐ and facility‐based distribution, while in West Africa, the focus is on secondary distribution among key populations [12].

Despite substantial self‐testing scale‐up, evidence about HIVST performance and impact when integrated into national health systems remains limited [13, 14]. Key questions persist: whether HIVST expands overall testing coverage or substitutes for conventional facility [15]; whether countries can ensure consistent large‐scale distribution; and whether linkage to treatment can be maintained at levels comparable to facility‐based testing [16, 17]. A recent systematic review reported high linkage among those with reactive self‐tests, but results varied significantly by distribution modality and setting [18].

This study leverages routinely collected programmatic data from eight countries in eastern and southern Africa (Kenya, Lesotho, Malawi, Mozambique, South Africa, Tanzania, Uganda and Zimbabwe) to address these gaps. We compare trends in conventional testing against HIVST, assess population testing rates and positivity, evaluate antiretroviral therapy (ART) linkage and investigate the stability of HIVST distribution over time. Our findings aim to inform efforts to optimize HIV testing strategies and accelerate progress towards epidemic control.

## 2 Methods

### 2.1 Data

We conducted a descriptive retrospective analysis using programmatic data from national HIV programmes in eight sub‐Saharan African countries with high‐quality routine data: Kenya, Lesotho, Malawi, Mozambique, South Africa, Tanzania, Uganda and Zimbabwe. These countries are situated in eastern and southern Africa, regions with the highest HIV prevalence globally, and have achieved some of the highest levels of knowledge of status among people living with HIV—with UNAIDS 2023 estimates ranging from 87% (95% credible interval [95% CrI]: 82%−97%) in Tanzania to 96% (95% CrI: 86%−98%) in Kenya [19]. Our primary objective was to characterize the first 3 years of HIVST scale‐up in each country. Data availability varied depending on when national programmes began reporting HIVST indicators and on data‐sharing processes with Ministries of Health: Kenya (Q4 2018–Q3 2022), Lesotho (Q1 2020–Q4 2023), Malawi (Q1 2019–Q3 2022), Mozambique (Q1 2020–Q1 2025), South Africa (Q1 2019–Q3 2022), Tanzania (Q1 2019–Q4 2023), Uganda (Q1 2020–Q3 2022) and Zimbabwe (Q1 2020–Q4 2023). The extended timeframe for Mozambique reflects the later start of the HIVST scale‐up in that country (2022). Data were initially extracted from national health information systems in April 2023; updated extracts were requested and received from some countries in February 2024. Table S2 provides details on data sources, including the Ministry offices that supplied data, reporting tools used and data availability.

Quarterly data on key HIV testing indicators were collected from national HIV programmes at the level commonly used for programme reporting: the second administrative level in seven of the eight countries and the first administrative level in Kenya—where counties have replaced provinces as the primary geographic subdivision since 2010. These differences in administrative levels result in varying numbers of subnational units between countries, which can, in turn, affect the extent of observed variation in testing outcomes. To ensure programmatic relevance and maintain consistency with national reporting structures, we analysed each country's data at the level at which it was collected. A supplementary table (see Table S1) summarizes the number of subnational units for each country.

Indicators collected per district per quarter included: the number of HIVST kits distributed, the number of facility‐based HIV tests conducted, the number of individuals newly diagnosed with HIV and the number of individuals initiating ART. Data were restricted to individuals aged 15 years and older, as HIVST is typically not distributed to younger age groups. These data reflect government programme activities and do not capture HIVST kits distributed through private sector or non‐governmental channels, which may be substantial in some settings. We used population estimates for 2020 at the sub‐national level from the WorldPop raster dataset [20] and aggregated these estimates to each administrative boundary using shapefiles from the Global Administrative Areas Database [21]. This allowed us to calculate testing rates per 1000 inhabitants.

### 2.2 Key Testing and Treatment Outcomes

Defining and evaluating testing effectiveness in this descriptive study involves several metrics. Population coverage refers to the proportion of a community tested over a specific period. We approximated this quantity for each testing modality by calculating the annual total number of tests conducted or kits distributed per inhabitant for each district. An important caveat regarding self‐testing rates is that we use numbers representing kits distributed rather than tests performed; actual use rates are unknown and likely vary by distribution modality. However, this metric provides insights into population testing coverage and allows for comparisons across districts of varying population sizes. We defined testing positivity as the proportion of people who tested positive out of 1000 tests performed. We approximated this metric by calculating the proportion of positive tests out of all tests performed in a district for each year. We defined ART linkage rate as the proportion initiating ART within 3 months among those who tested positive. We approximated this by dividing the total number of new ART initiations by the total number of new HIV diagnoses, for each district and year. This proxy measure can exceed 100% in some districts because new ART initiations may include individuals who were diagnosed in other districts, those re‐initiating ART after disengagement or individuals who received a positive HIVST result from non‐programme sources (e.g. private sector) and subsequently sought care at public facilities. Finally, we developed a stability metric to assess the consistency in volume of HIVST distribution. We calculated the coefficient of variation, a standardized measure of dispersion [22], as the ratio of the standard deviation ($SD_{i[c]}$) to the mean number of HIVST kits distributed by district ($\bar{x_{i[c]}}$), over the last four quarters of data in each country *c*, once countries had scaled up HIVST distribution. We created a sustainability metric as one minus the coefficient of variation:

$$\;CV_{i\left[c\right]}=\frac{SD_{i\left[c\right]}}{\bar{x_{i\left[c\right]}}}$$

$$S_{i\left[c\right]}=1-CV_{i\left[c\right]}$$

Higher stability scores ($S_{i[c]}$) imply lower coefficient of variations ($CV_{i[c]}$), and hence indicate greater consistency in HIVST distribution for a given district *i*, while lower or negative stability scores indicate higher dispersion, and therefore, lower consistency.

This study utilized aggregated, de‐identified programmatic data without personal identifiers. As no individual‐level data were collected or accessed, informed consent was not required, and a consent waiver was not sought. Ethical approval was not required. Data‐sharing agreements with national programmes were established and respected, and data were handled securely in accordance with relevant guidelines and regulations. All data included and analysed belong to the respective Ministries of Health.

## 3 Results

### 3.1 National‐Level HIV Conventional Testing Volumes and Self‐Test Kits Distributed Over Time

**FIGURE 1:**
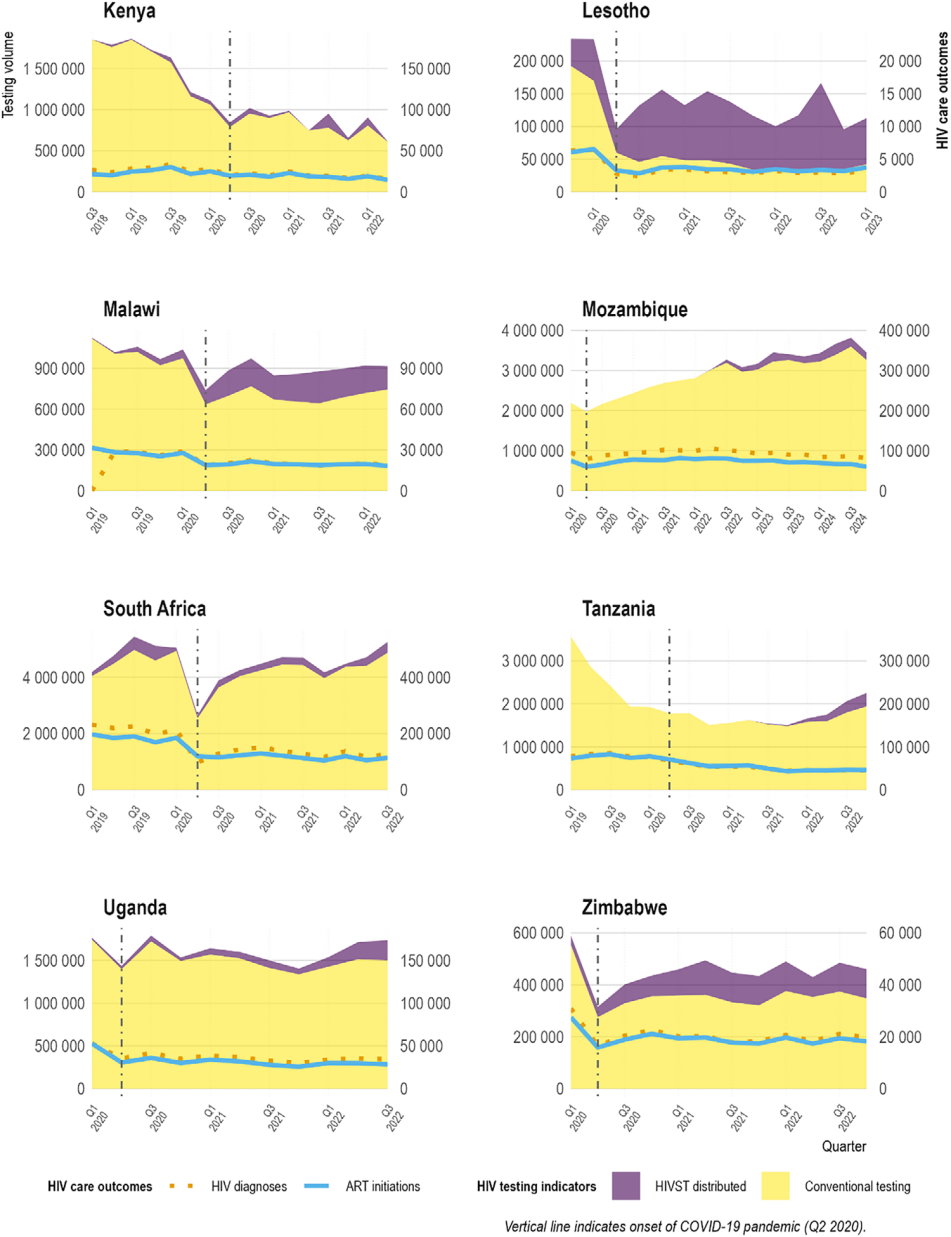
Number of conventional testing and HIV self‐testing (HIVST) kits distributed, and number of new HIV diagnoses and ART initiations reported in each country. Note that due to the vast differences in scale between HIV testing indicators and HIV care outcomes, two different y‐axes are used. The left y‐axis is used for the HIV testing indicators, while the right y‐axis is used for the HIV care outcomes.

During the study period from the first quarter (Q1) of 2019 (Q1 2020 in Mozambique) to the fourth quarter (Q4) of 2023 (Q4 2024 in Mozambique), we observed significant variations in trends in total HIV testing volumes across the eight countries (Figure 1). The contribution of HIVST to total testing varied substantially: by the most recent quarter of available data, HIVST accounted for 63% of total testing in Lesotho, 25% in Zimbabwe, 19% in Malawi, 14% in Tanzania, 14% in Uganda, 7% in South Africa, 6% in Mozambique and less than 1% in Kenya (Figure S1). In Kenya and Tanzania, large declines in facility‐based HIV tests were observed for several quarters before the onset of the COVID‐19 pandemic (in Q2 2020) and continued throughout the study period, while the number of HIV self‐tests increased modestly. In Kenya, the total number of conventional facility‐based HIV tests dropped from 1.85 million in Q1 2019 to 609,600 in Q3 2022, corresponding to a 67% decrease in conventional testing volume. The total HIV testing volumes (conventional and self‐tests combined) decreased by 66% over that period. Similarly, in Tanzania, conventional HIV testing decreased by 46% from about 3.56 million tests in Q1 2019 to approximately 1.93 million tests in Q4 2022, and the total HIV testing volumes decreased by 37%. In Lesotho, the sharp 78% decline in conventional tests, from about 192,400 in Q4 2019 to 42,000 conventional tests in Q1 2023, was partially offset by a large increase in quarterly HIVST distribution—reaching 112,750 kits distributed in Q1 2023.

South Africa's conventional testing volumes rebounded substantially after Q2 2020 to reach 4.87 million in Q3 2022—a 21% increase from 4.04 million in Q1 2019. In Malawi, despite a 33% decrease in the conventional tests over the study period, a significant 25‐fold increase in HIVST kit distribution, from 6600 in Q1 2019 to 202,596 in Q2 2022, reduced the decrease in total testing volumes to only 18%. Zimbabwe also increased its HIVST distribution from 36,800 in Q1 2020 to 113,000 kits in Q1 2023, mitigating a 37% decrease in conventional tests. Finally, Uganda maintained relatively stable testing volumes throughout the study period (−1%), while Mozambique exhibited a continued expansion of both HIVST and conventional testing, resulting in a 57% increase between Q1 2020 and Q4 2024.

### 3.2 National‐Level Population HIV Testing Rates Over Time

**FIGURE 2:**
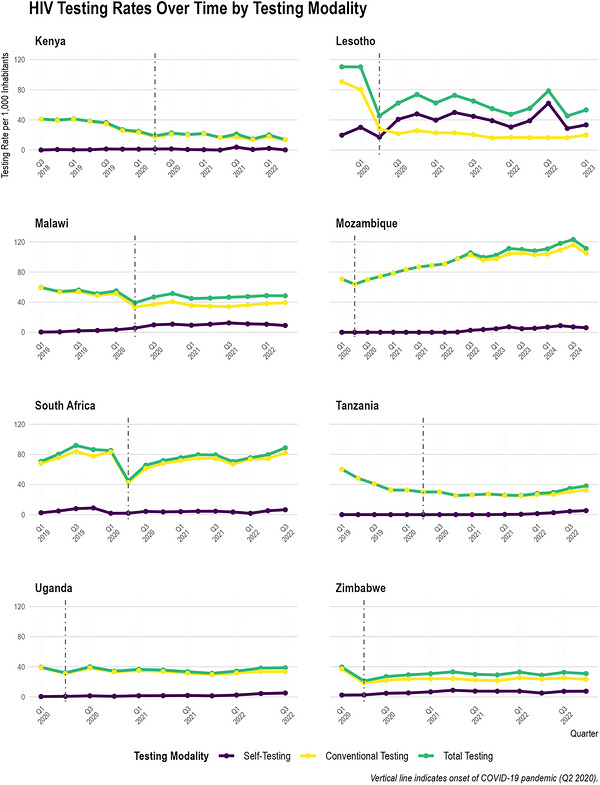
HIV testing rates per 1000 inhabitants over time by testing modality in each country. Self‐testing rates represent HIVST kits distribution per 1000 inhabitants, conventional testing rates represent facility‐based testing and total testing rates represent the sum of both modalities. The vertical dashed line marks Q2 2020, representing the onset of the COVID‐19 pandemic. Note that different countries may have different data availability periods, hence the use of free x‐axis scales to accommodate varying start and end quarters.

Standardizing the quarterly testing rates facilitates direct comparisons between countries of varying population sizes (Figure 2). In Lesotho, total testing rates decreased from 110 per 1000 inhabitants in the first quarter of 2020 to 53.2 per 1000 by 2023. This decline in total testing was primarily driven by a sharp reduction in conventional testing rates from 90.8 to 19.8 per 1000, juxtaposed with a substantial increase in self‐testing rates from 19.7 to 33.3 per 1000. South Africa maintained high testing rates: from a total testing rate of 70.7 per 1000 inhabitants in 2019 to 88.7 per 1000 by 2022. This increase was fuelled by conventional testing, which rose from 68.2 to 82.2 per 1000, while self‐testing increased modestly from 2.47 to 6.50 per 1000. In Mozambique, total testing rates increased from 32.7 per 1000 inhabitants in the first quarter of 2020 to 51.5 per 1000 by 2024. This increase was primarily driven by conventional testing, which climbed from 32.7 to 45.6 per 1000, alongside a rise in self‐testing from 0 to 5.94 per 1000.

### 3.3 Variations in Testing Positivity for Districts With and Without Self‐Test Kits Distribution

**FIGURE 3:**
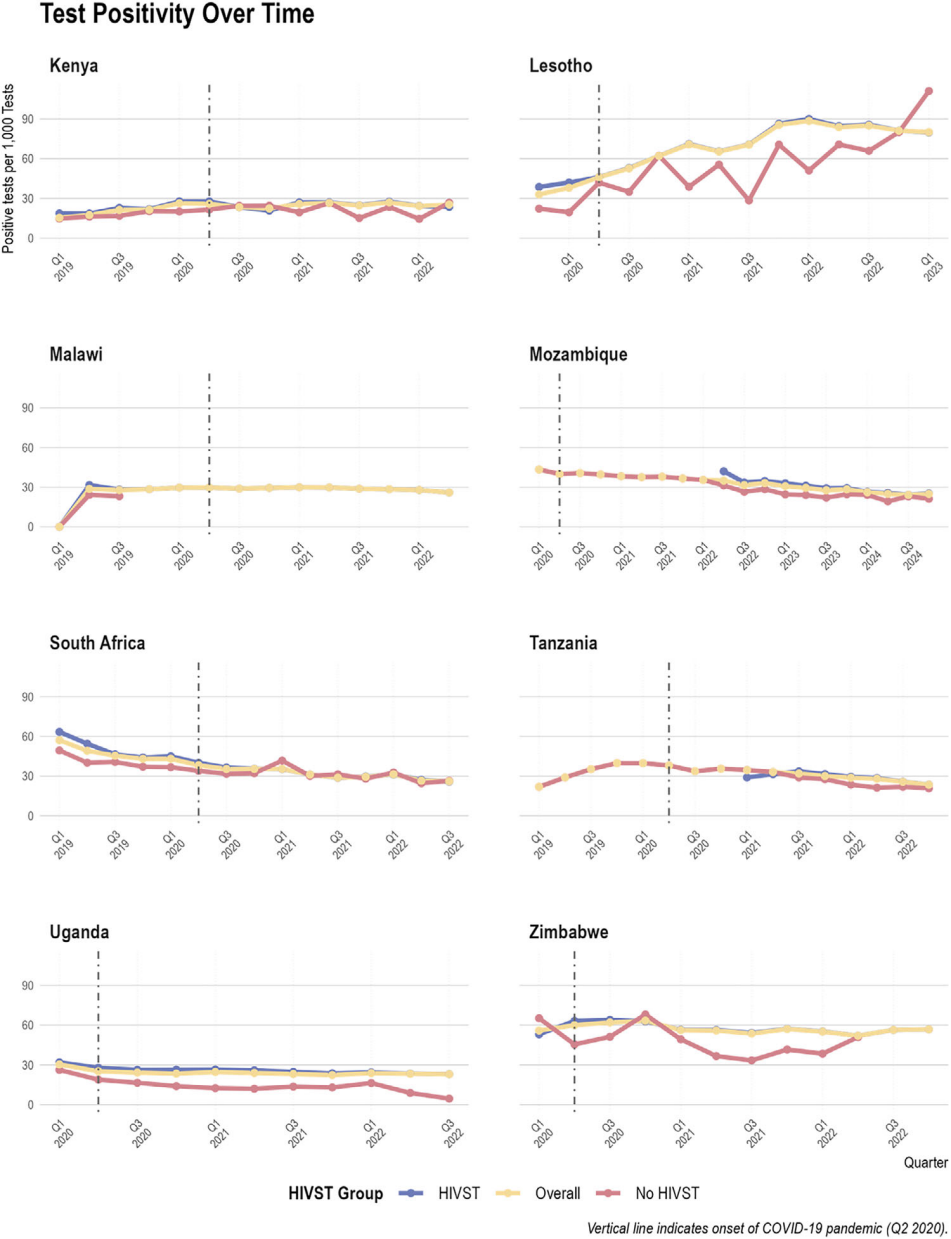
HIV test positivity rates (positive tests per 1000 tests) over time by HIVST group in each country. Districts are classified as “HIVST” (received HIV self‐testing kits), “No HIVST” (did not receive HIV self‐testing kits) or “Overall” (aggregate across all districts regardless of HIVST status). The vertical dashed line marks Q2 2020, representing the onset of the COVID‐19 pandemic, which significantly impacted HIV testing services globally.

Overall positivity stayed relatively stable or declined slowly in most settings (Figure 3), except for Lesotho and Malawi, both of which showed a slight increase in median positivity over the study period: rising from 18 to 27 positive tests per 1000 tests in Lesotho between 2019 and 2022, and from 23 to 29 positive tests per 1000 tests in Malawi between 2019 and 2023. Disaggregating by HIVST status shows that in most quarters and countries, districts distributing HIVST recorded higher positivity rates than those yet to introduce HIVST. For example, in Kenya in 2019, districts that distributed HIVST had a median positivity of approximately 20 per 1000 tests, compared to 17 per 1000 in districts without HIVST; in Zimbabwe in 2020, the comparison was 16 versus 13 per 1000 tests and, in Mozambique in 2022, it was 30 versus 28 per 1000 tests. However, it is important to note that these observed differences likely reflect confounding, as HIVST distribution was often prioritized for districts with historically higher positivity. Therefore, we did not apply formal statistical tests to compare positivity by HIVST distribution status.

Comparisons between districts revealed considerable within‐country variability, particularly in Mozambique, where positivity in 2022 ranged from fewer than 10 to over 100 new HIV diagnoses per 1000 tests (Figure S2). Similar, though somewhat narrower, variations are evident in Malawi and Zimbabwe. In contrast, Uganda showed more modest variation, with most districts clustered near the national average.

### 3.4 Variations in ART Linkage Rates for Districts With and Without Self‐Test Kits Distribution

**FIGURE 4:**
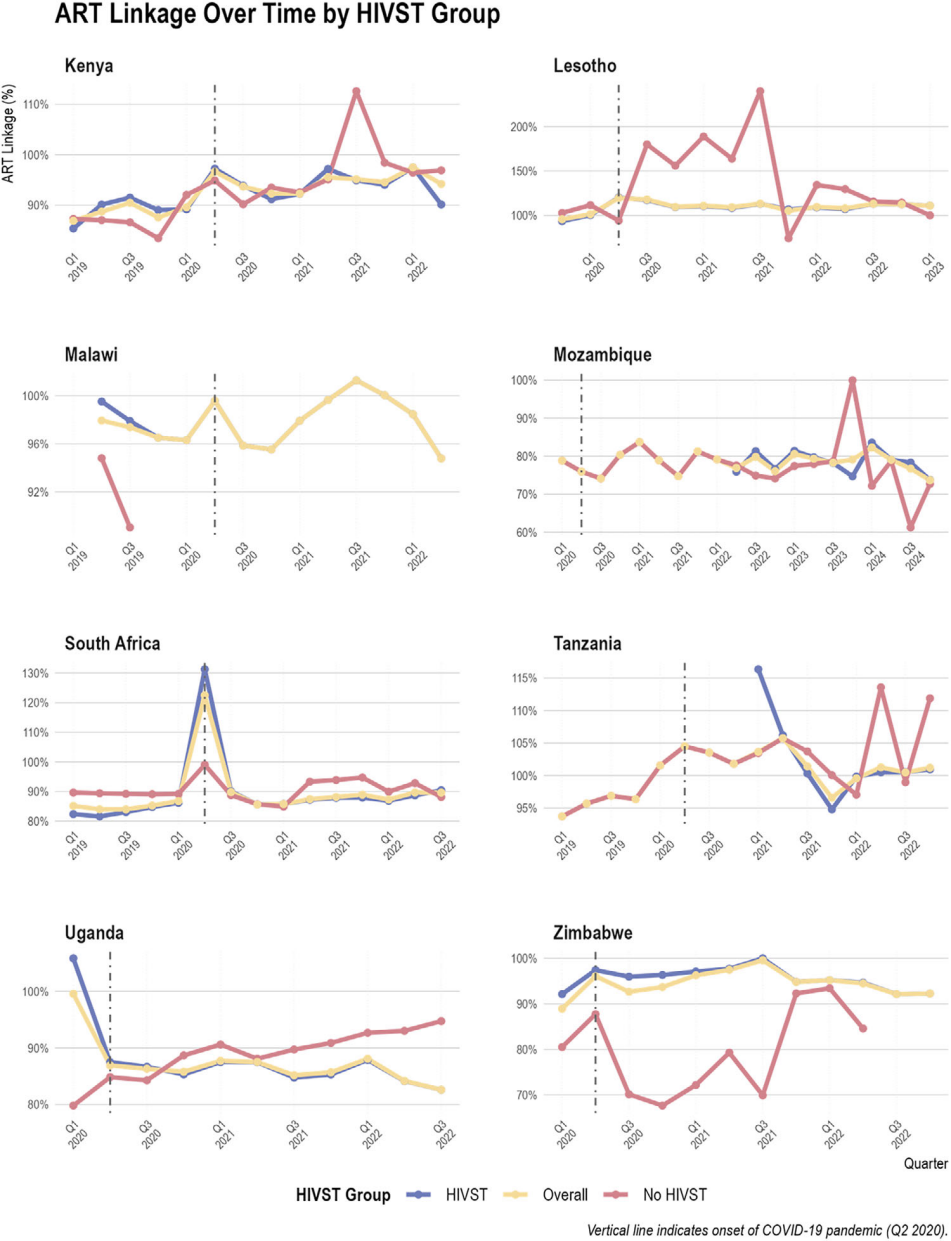
ART linkage rates (percentage of newly diagnosed HIV‐positive individuals who initiated antiretroviral therapy) over time by HIVST group in each country. Districts are classified as “HIVST” (received HIV self‐testing kits), “No HIVST” (did not receive HIV self‐testing kits) or “Overall” (aggregate across all districts regardless of HIVST status). The vertical dashed line marks Q2 2020, representing the onset of the COVID‐19 pandemic.

ART linkage rates showed relative stability across years and countries (Figure 4). In Kenya, for example, median linkage rates in districts where HIVST was implemented hovered between 90% in 2019 and 94% in 2022, and in non‐HIVST districts, the medians remained similarly high (86.8% in 2019, 96.7% in 2022). Similarly, Uganda's 2020 linkage medians for HIVST and non‐HIVST districts (87.1% vs. 84.6%) suggest minimal differences between groups.

In general, there were no major, consistent patterns of differences in linkage rates based on whether districts distributed HIVST. In Zimbabwe, while there was a noticeable gap in 2020 (HIVST median 96.2% vs. non‐HIVST median 75.3%), the difference narrowed by 2022 (93.5% vs. 89.0%). A similar pattern was seen in Malawi, Mozambique and South Africa, where median rates for HIVST and non‐HIVST districts generally differed by only a few percentage points. A notable pattern across countries was that several districts reported linkage rates exceeding 100%. This likely reflects methodological limitations of the proxy measure: new ART initiations may include individuals diagnosed in other districts who sought care locally, those re‐initiating ART after prior disengagement or individuals who tested positive using privately obtained HIVST and subsequently presented to public facilities for care. In Lesotho, Tanzania and Mozambique, most districts reported linkage rates above 100%.

### 3.5 Stability of HIVST Distribution

**FIGURE 5:**
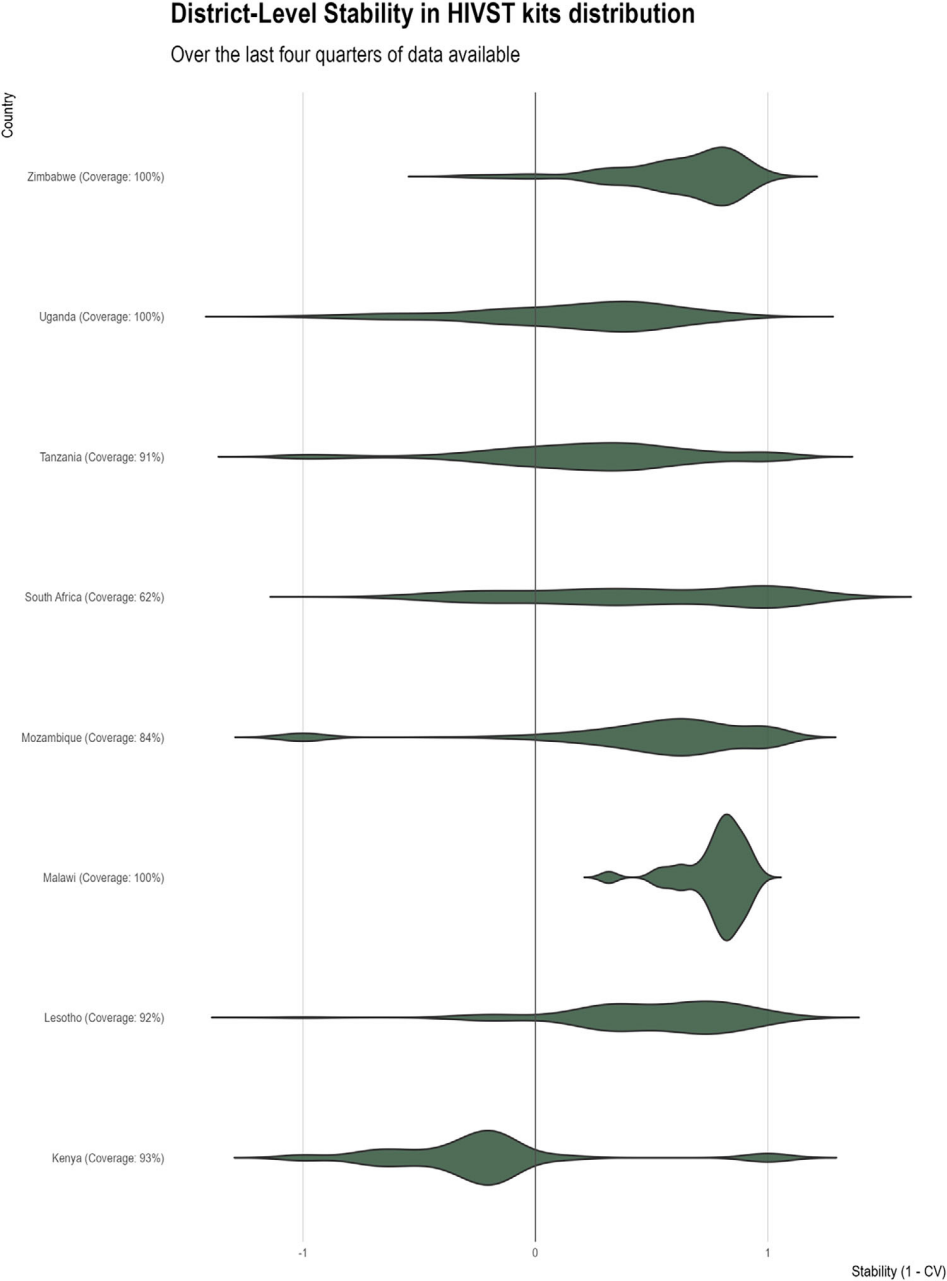
Stability metrics for HIV self‐testing distribution across countries based on the last four quarters of available data. This figure shows country‐level stability scores calculated as 1 minus the median coefficient of variation (CV) of HIVST distribution across districts. It displays the distribution of district‐level stability scores as violin plots, with country labels indicating the percentage coverage of HIVST distribution. The horizontal reference line at y = 0 helps distinguish between positive and negative stability values. Higher stability scores indicate more consistent HIVST distribution patterns across districts within each country.

The stability of HIVST distribution varied substantially, both on average across countries and within countries (Figure 5). Malawi showed notably high stability, with scores clustered near 0.95 and very few districts dipping below 0.50. This suggests that Malawi has achieved both wide coverage and highly consistent quarterly distribution. Zimbabwe also exhibited a relatively strong performance, though its distribution was somewhat broader, ranging from about 0.05 at the low end up to around 1, implying that while many districts had stable HIVST supply, some still experienced moderate fluctuations over time. In Lesotho, the stability scores appeared bimodal, with one cluster centred near 0.75 and another around 0.30, indicating two subgroups of districts with different degrees of supply regularity. Mozambique presented a unimodal yet left‐skewed distribution, suggesting that although most districts achieved moderate stability (centred near 0.45), some experienced more frequent or larger quarterly fluctuations. In contrast, Kenya occupied the lower end of the spectrum, with a left‐skewed distribution centred around −0.15 and extending to some distinctly negative values. This pattern indicates a subset of districts with particularly erratic HIVST supply from quarter to quarter, while others showed more moderate stability.

## 4 Discussion

This study describes the real‐world implementation of HIVST across eight African countries from 2019 to 2023. Our findings underscore substantial heterogeneity in how HIVST is integrated into national testing strategies, highlighting the importance of understanding distinct local contexts and policy decisions. Importantly, the analyses are descriptive and based on routinely collected programme data; they characterize patterns over time and across districts but do not establish causal effects.

Our analysis reveals significant variation in HIV testing volumes, with some countries maintaining high conventional testing (South Africa, Uganda) and others experiencing large decreases (e.g. Lesotho, Kenya, Tanzania). South Africa's testing volumes rebounded quickly after COVID‐19, likely due to strong domestic financing [23, 24]. Across eastern and southern Africa, the study period also coincided with declining HIV incidence, progress towards HIV testing and treatment targets, alongside shifts in donor guidance, including PEPFAR's growing emphasis on testing yield and differentiated testing approaches. These factors, together with resource constraints, likely influenced programmes in several countries to reduce overall testing volumes and reorient testing efforts towards higher‐risk populations and priority locations. HIVST has been used as a complement to conventional testing, particularly where traditional testing has declined. In Lesotho and Malawi, large‐scale HIVST distribution coincided with reductions in conventional testing, though these patterns reflect programmatic choices rather than evidence that HIVST “compensated for” declines. Maintaining adequate testing coverage remains essential if countries are to achieve and sustain low HIV acquisition and end AIDS by 2030 with available resources. Testing volume reductions may lead to modest short‐term financial gains but to slower progress towards epidemic control, which would in turn lead to larger long‐term HIV programme costs [25]. Lesotho showed the most dramatic shift, with HIVST positioned as a facility‐based tool since 2019, alongside community distribution models [26], to save health workers’ time. Previous studies showed early HIVST introduction was cost‐effective and high‐impact [27], but further engagement is needed to understand the drivers of the transition to HIVST over alternative testing approaches in Lesotho and to assess whether similar scale‐up could be operationalized and achieve comparable results elsewhere.

Districts receiving HIVST often had higher observed positivity, consistent with being prioritization to higher‐burden in line with PEPFAR's focus on testing yield. However, without formal statistical comparisons, these descriptive patterns should not be interpreted as evidence that HIVST itself increased positivity. In most countries, we observed declining test positivity as programmes advanced towards the first 95 targets [28]. This may also reflect HIVST's growing role in HIV prevention supporting pre‐exposure prophylaxis (PrEP) and post‐exposure prophylaxis (PEP) access but generating high volumes of negative self‐tests [29, 30]. With only ∼2.5 million of the 10 million PrEP initiations targeted for 2025 achieved [31], HIVST offers a practical way to close that gap [32]. Programmes should track how this prevention‐focused use reshapes positivity metrics and their interpretation.

ART linkage rates varied widely within countries and were generally similar in HIVST and non‐HIVST districts (except in Zimbabwe, where HIVST districts performed better). Several districts exceeded 100% linkage, likely reflecting cross‐district care‐seeking, individuals re‐initiating ART after disengagement or people who tested positive using privately obtained HIVST and subsequently sought care at public facilities. These patterns highlight the need to interpret routine linkage metrics cautiously.

The stability of HIVST distribution varied across countries. Lesotho distributed the highest volume of HIVST kits relative to its population; however, its stability scores were bimodal, indicating that while some districts achieved relatively consistent distribution patterns over time, others experienced more pronounced fluctuations in supply. Mozambique also showed high consistency scores and gradually increasing HIVST volumes, reflecting the potential for further scale‐up in the future. Malawi and Zimbabwe exhibited both the highest consistency and coverage of HIVST across districts; however, self‐testing still represents only a fraction of total testing volumes, suggesting that while both programmes are stable, they have not followed the substitution trend seen in Lesotho. Additionally, while not recorded explicitly, anecdotal evidence of stock‐outs was reported by countries, which may have affected some of the results and consistency trends. For example, in Lesotho, intermittent stock‐outs of HIVST were flagged by district teams and correlated with abrupt dips in both self‐testing volume and HIV positivity. Lessons can be applied from these experiences and better leveraged to develop robust supply chains, strong political commitment and ongoing programmatic support.

This study has several limitations inherent to using programmatic data across multiple countries. First, we used aggregated district‐level data constraining subgroup analyses—for instance, pregnant women attending antenatal care, key populations at higher risk of HIV acquisition and individuals initiating PrEP are encouraged to test more frequently. As a result, our testing coverage metric serves as a proxy and may overestimate the true proportion of the population tested. Moreover, in most countries, testing data could not be consistently disaggregated by age, sex or key population, further limiting our ability to assess equity in who was reached by conventional testing and HIVST. Second, because HIVST use is anonymous, we used proxy measures for ART linkage that cannot distinguish where patients were diagnosed or whether they were re‐initiating therapy, which previous studies have shown is common due to privacy concerns, perceived quality of care or ease of access [33, 34]. This likely explains why we observed linkage rates larger than 100% in some districts. Third, our data capture only government‐distributed kits, potentially underestimating HIVST coverage in countries with substantial private‐sector or donor‐funded pharmacy‐based initiatives, such as Kenya and Tanzania [35, 36]. Fourth, reporting completeness varied across countries and quarters, affecting the reliability of our consistency metric. We did not apply formal adjustments for incomplete reporting and instead relied on the best available national programme datasets; comparisons over time and between countries should, therefore, be interpreted with appropriate caution. Fifth, the temporal coverage of our data varied across countries, reflecting differences in when national programmes began systematically reporting HIVST indicators and in the processes required to access programmatic data from each Ministry of Health. While our goal was to characterize the first 3 years of HIVST scale‐up in each setting, these time windows do not align across countries—meaning that the observed patterns reflect different moments in the evolving global HIV response. This lack of temporal harmonization should be considered when interpreting differences in scale‐up trajectories. Finally, as a descriptive study, our analysis does not establish causal relationships; districts receiving higher HIVST volumes may have been prioritized due to programmatic targets or higher HIV burden.

Despite these limitations, this study leverages comprehensive, national‐level data across eight countries, with district‐level quarterly granularity enabling detailed temporal and spatial analyses. The methodologies and insights presented here can be routinely implemented by countries as an additional automated layer on their health information system. However, progress faces new challenges from recent policy and financial shifts, reducing global health funding.

## 5 Conclusions

This study provides insights into the real‐world HIVST implementation in eastern and southern Africa. The sustained distribution of HIVST kits in several countries demonstrates the feasibility of integration into national strategies, though further research is needed to quantify effects on outcomes. By complementing conventional testing, HIVST can contribute to achieving global targets. Countries should prioritize investing in systems to collect and analyse routine HIVST data to optimize programming.

## Author Contributions

AA, CCJ, AS‐F and JL developed the research project. AA and AS‐F reviewed data inputs. AA developed the analyses. All authors contributed to the interpretation of the study results. AA and CCJ wrote the first draft of the manuscript. All authors critically edited the manuscript for intellectual content and approved the final version of the manuscript for publication.

## Conflicts of Interest

The authors declare no conflicts of interest.

## Supporting information




**Figure S1**: Distribution of conventional testing and HIV self‐testing (HIVST) as a proportion of the total testing volume, over time and by country.
**Figure S2**: Number of HIV self‐test kits distributed (left) and conventional tests performed (right) per 1000 inhabitants, in each district for the most recent quarter of data available.
**Table S1**: Level of analysis for each country, and the corresponding number of subnational units at that level.
**Table S2**. Data sources by country.
*Notes*: All data were obtained through formal data‐sharing agreements facilitated by WHO. DHIS2 (District Health Information Software version 2) is the underlying platform for all national health management information systems, though local implementations may have country‐specific names (e.g. SISMA).

## Data Availability

The data underlying this study were obtained from national HIV programmes through data‐sharing agreements with the respective Ministries of Health. These data are not publicly available due to the terms of the data‐sharing agreements, which restrict redistribution. Requests for data access should be directed to the relevant Ministry of Health in each country. Aggregated summary statistics are available from the corresponding author upon reasonable request.
