# Supplementary material for: Scaling Up HIV Self‐Testing in Africa: Insights From National Programmatic Data in Eight Countries
Source: J Int AIDS Soc. 2026 Apr 29;29(5):e70120. doi: 10.1002/jia2.70120 (PMC13125956; doi:10.1002/jia2.70120)
Supplement: Supplementary file 1 — Figure S1: Distribution of conventional testing and HIV self‐testing (HIVST) as a proportion of the total testing volume, over time and by country. Figure S2: Number of HIV self‐test kits distributed (left) and conventional tests performed (right) per 1000 inhabitants, in each district for the most recent quarter of data available. Table S1: Level of analysis for each country, and the corresponding number of subnational units at that level. Table S2. Data sources by country. Notes: All data were obtained through formal data‐sharing agreements facilitated by WHO. DHIS2 (District Health Information Software version 2) is the underlying platform for all national health management information systems, though local implementations may have country‐specific names (e.g. SISMA). [file JIA2-29-e70120-s001.docx]

**Supplementary Figures**


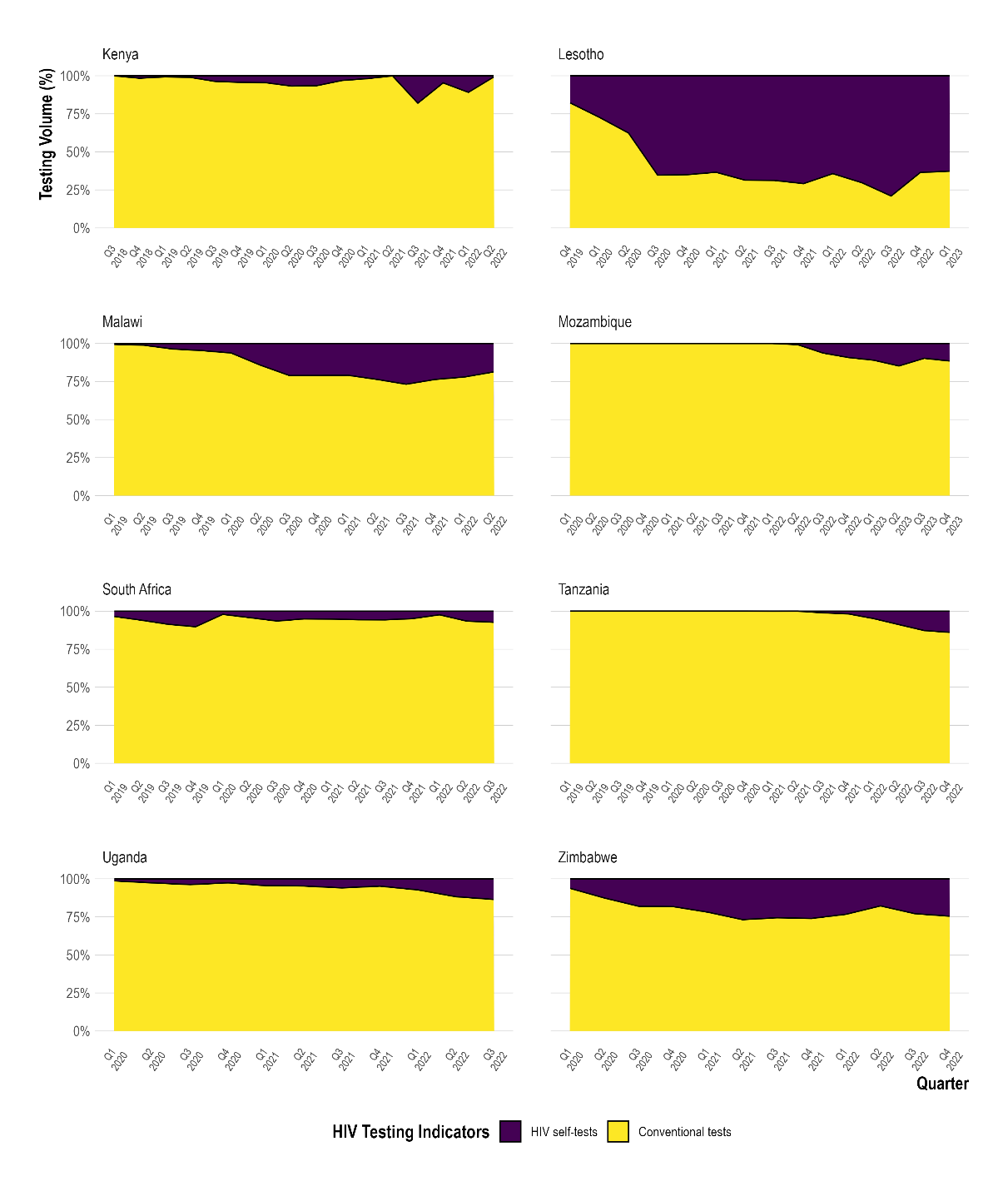


**Figure S1: Distribution of conventional testing and HIV self-testing (HIVST) as a proportion of the total testing volume, over time, and by country**.


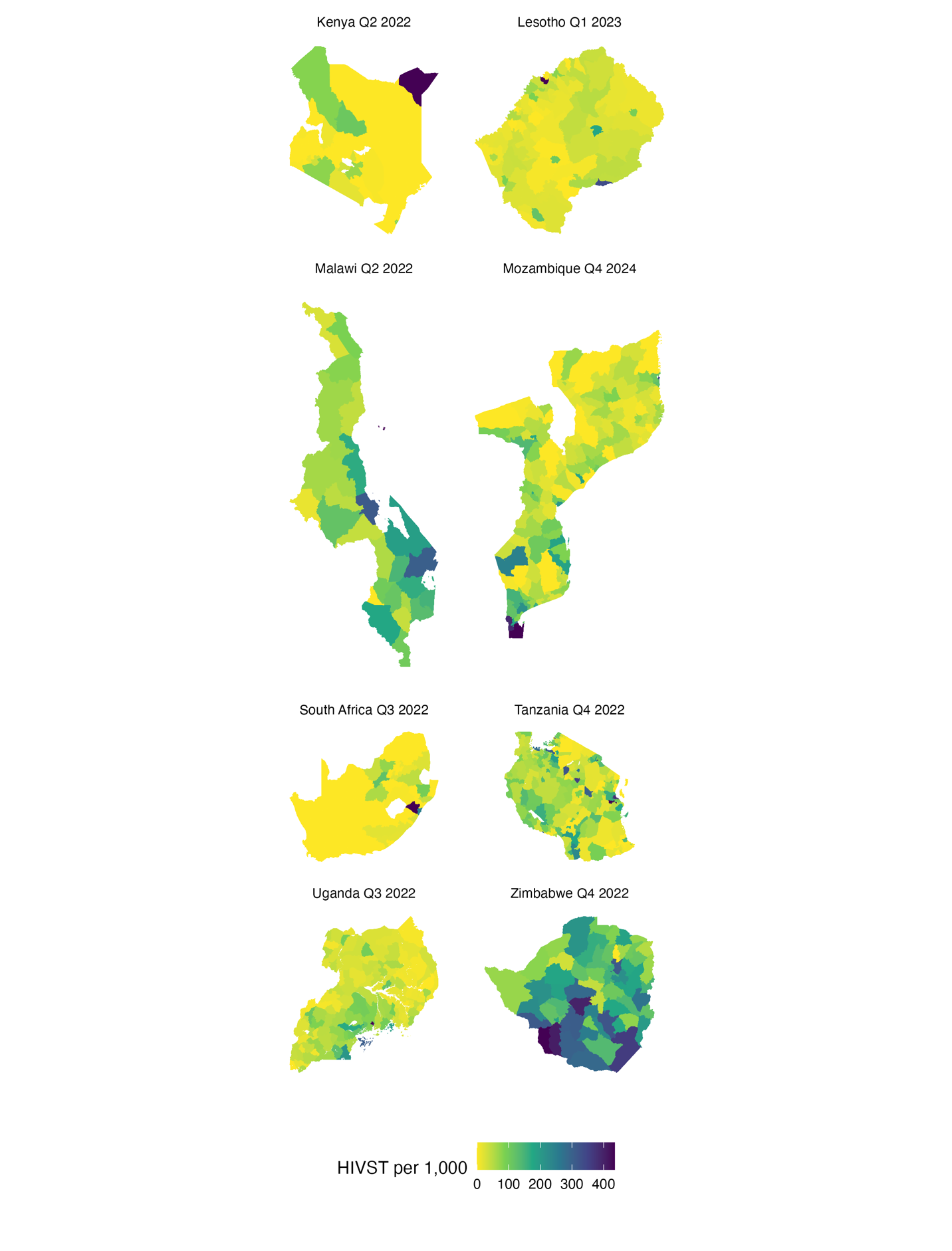

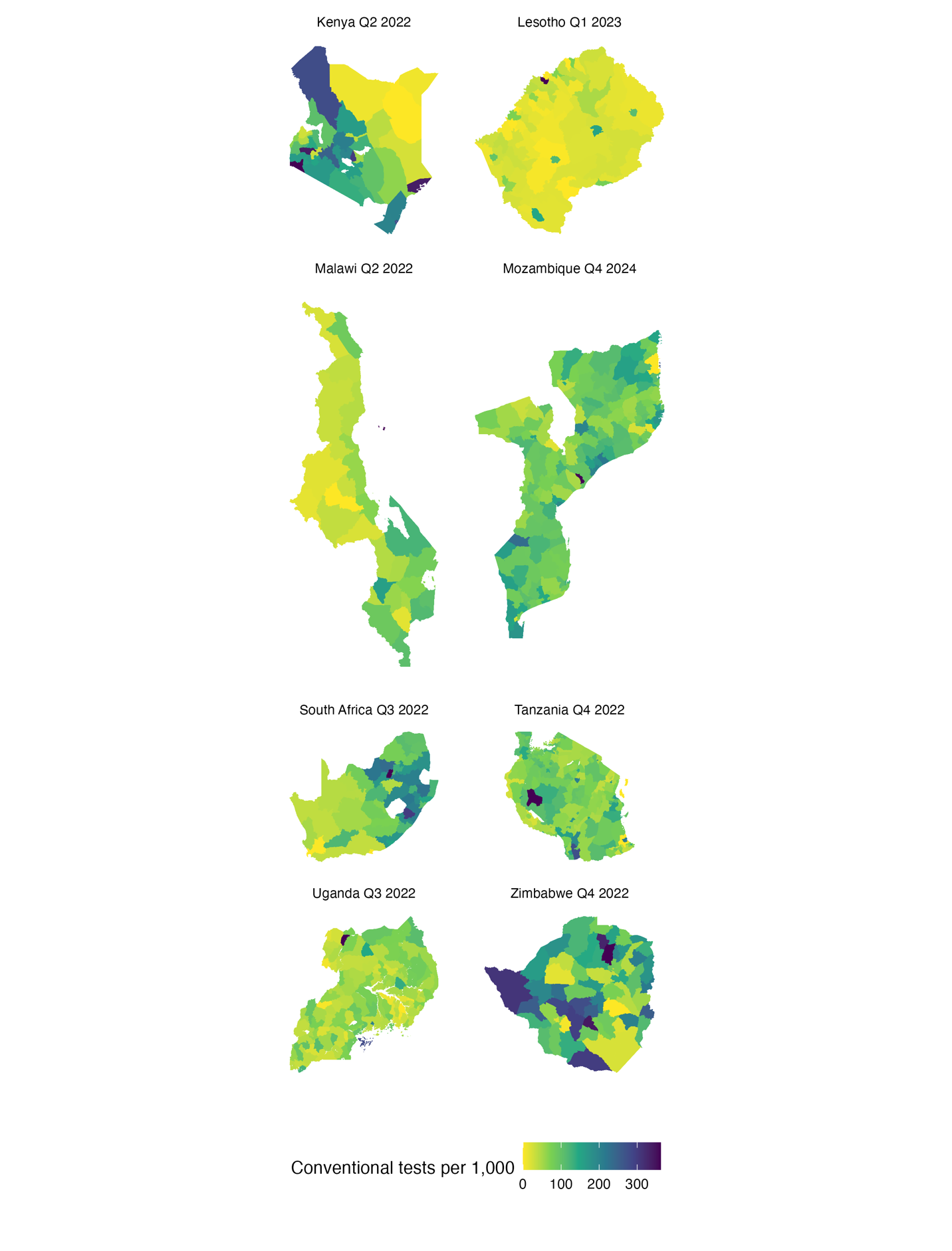


**Figure S2: Number of HIV self-test kits distributed (left) and conventional tests performed (right) per 1,000 inhabitants, in each district for the most recent quarter of data available.**

**Table S1: Level of analysis for each country, and the corresponding number of subnational units at that level.**

| Country | Level of Analysis | No. of Subnational Units |
| --- | --- | --- |
| Kenya | Counties | 52 |
| Lesotho | Constituencies | 80 |
| Malawi | Districts | 28 |
| Mozambique | Districts | 161 |
| South Africa | Districts | 52 |
| Tanzania | Districts | 169 |
| Uganda | Districts | 135 |
| Zimbabwe | Districts | 64 |

**Table S2. Data sources by country**

| **Country** | **Ministry Office / Programme** | **Reporting Platform** | **Local System Name** | **Data Publicly Available** |
| --- | --- | --- | --- | --- |
| **Kenya** | **Ministry of Health, National AIDS and STI Control Programme (NASCOP)** | **DHIS2** | **Kenya Health Information System (KHIS)** | **No** |
| **Lesotho** | **Ministry of Health, National AIDS Control Programme** | **DHIS2** | **DHIS2** | **No** |
| **Malawi** | **Ministry of Health and Population, Directorate of HIV, STI and Viral Hepatitis** | **DHIS2** | **Malawi HMIS** | **No** |
| **Mozambique** | **Ministerio da Saude, Programa Nacional de Controlo de ITS/HIV/SIDA** | **DHIS2** | **SISMA (Sistema de Informacao de Saude para Monitoria e Avaliacao)** | **No** |
| **South Africa** | **National Department of Health, National AIDS Programme** | **DHIS2** | **DHIS2/ TIER.Net** | **No** |
| **Tanzania** | **Ministry of Health, National AIDS Control Programme (NACP)** | **DHIS2** | **DHIS2** | **No** |
| **Uganda** | **Ministry of Health, AIDS Control Programme** | **DHIS2** | **Uganda eHMIS** | **No** |
| **Zimbabwe** | **Ministry of Health and Child Care** | **DHIS2** | **DHIS2** | **No** |

**Notes:** All data were obtained through formal data-sharing agreements facilitated by WHO. DHIS2 (District Health Information Software version 2) is the underlying platform for all national health management information systems, though local implementations may have country-specific names (e.g. SISMA).
